# A Pilot Qualitative Investigation of Stakeholders’ Experiences and Opinions of Equine Insect Bite Hypersensitivity in England

**DOI:** 10.3390/vetsci5010003

**Published:** 2018-01-09

**Authors:** Hannah R. Lomas, Philip A. Robinson

**Affiliations:** Department of Animal Production, Welfare and Veterinary Sciences, Harper Adams University, Newport, Shropshire TF10 8NB, UK

**Keywords:** equine insect bite hypersensitivity, sweet itch, stakeholder opinions and experiences, semi-structured interviews, qualitative research

## Abstract

Equine insect bite hypersensitivity (IBH), commonly known as sweet itch or summer eczema, is a frustrating recurrent skin disease in the equine industry involving an immune reaction to the bites of *Culicoides* spp. midges. To investigate the impact of IBH in the field, an exploratory pilot study was conducted with equine stakeholders in one region of central England. Nine semi-structured, face-to-face interviews were conducted with horse owners and an equine veterinarian. The aim was to gain an understanding of experiences with IBH, and to gauge opinions on the value of the various management strategies horse owners use to control IBH. Awareness of IBH was generally high, particularly in those individuals who had previous experience with the condition. Those with previous experience of IBH commented on the significant effect on daily routines, and the associated cost implications. Most participants supported an integrated approach to hypersensitivity management, and this most commonly involved a combination of physical barriers and chemical repellents, but sometimes included feed supplementation. Overall, attitudes towards IBH suggested that the condition is a notable welfare and economic concern for stakeholders, but veterinary involvement tended to only be in more severe cases. Further research is required in the future to improve understanding, management and potential treatment of this condition.

## 1. Introduction

Equine insect bite hypersensitivity (IBH), also commonly known as sweet itch or summer eczema, is an IgE-mediated, seasonal, and pruritic dermatosis of the horse [1]. It is a frustrating recurrent problem experienced by many horse owners as one of the most common allergic reactions affecting a wide range of horse and pony breeds [2]. The hypersensitivity is caused by the allergens contained in the saliva of the *Culicoides* spp. biting midges, and possibly other biting insects such as *Simulidae* (black flies) [3,4]. This hypersensitivity leads to self-inflicted trauma that can result in alopecia, bleeding and crusting of the skin [5]. Chronically affected animals can also develop skin rugae at the base of the mane and tail due to intradermal oedema [6].

The *Culicoides* midge is most active during dawn and dusk in hot and humid conditions [4]. The greatest number of midges is present where grazing is bordered by high hedging or wooded areas [4] and they breed in areas such as water troughs and stagnant water ponds [7]. Currently, a combination of physical barriers and chemical repellents have been suggested to be the most effective management strategies to control the *Culicoides* midge and subsequent IBH [4], as there is currently no cure for the condition [1]. Hallamaa (2009) [8] has suggested IBH to be one of the equine diseases that most commonly impairs the quality of life of horses, and is therefore a welfare issue with the potential to cause significant economic cost for horse owners with affected animals. This makes IBH a serious condition that requires continued research to establish effective prevention and treatment for the condition. This paper begins by reviewing some of the published literature on IBH to set the challenge of controlling IBH in context, and then proceeds to present the findings of equine stakeholder interviews concerning their views on the condition and its prevention and management.

### 1.1. Aetiology and Risk Factors for IBH

IBH develops due to an allergic reaction when the horse’s immune system overreacts to the saliva of *Culicoides* acquired from bites [9]. These bites invoke predominantly type 1 hypersensitivity (within six hours of the bite) and type 4 (delayed) hypersensitivity reactions [10]. Multiple midge salivary proteins, including, for example, maltase and hyaluronidase, have been demonstrated to be allergens associated with IBH [1]. Although proteins in midge saliva are thought to be the main allergens, Hellberg et al. (2009) [3] has also demonstrated shared IgE-binding salivary proteins in both *Culicoides* and *Simulidae* (black flies), and both could therefore be responsible for the hypersensitivity reactions. When the midge’s saliva enters the horse it induces rapid cross-linking of receptor-bound IgE, and subsequent release of inflammatory mediators [9].

*Culicoides* spp. are poor fliers, making them less prevalent in exposed and windy areas with well-draining soil, and conversely a greater number are present where grazing is bordered with high hedging or wooded areas. The breeding site of *Culicoides* is typically in standing water, and the larvae are often found there [11]. *Culicoides* is most active around dawn and dusk and in humid conditions, making these times when the horse will be more likely to be bitten, and when management is essential [4].

IBH does not occur in Iceland due to the lack of *Culicoides*, however, it has been found to be present in Icelandic horses that have been imported into areas where the *Culicoides* midge is present. A study by Björnsdóttir et al. (2006) [12] discovered that over 50% of Icelandic horses imported into the European continent from Iceland developed IBH within two years or more when exposed to heavily infested *Culicoides* areas, but Icelandic horses born outside of Iceland have a lower or similar frequency of IBH as other breeds in Europe. Although the genes that are linked to IBH have not been identified, some studies have found that certain breeds are more susceptible than others. Eriksson et al. (2008) [13] studied Swedish-born Icelandic horses and found a prevalence of IBH of 8% in the breed, and Schurink et al. (2012) [14] identified several genomic regions associated with IBH in both Shetland Pony mares and Icelandic horses. Schurink et al. (2014) [15] also found that Shetland Ponies had a significantly higher IgE reactivity against most *Culicoides obsoletus* complex allergens when compared with Icelandic horses. Horse breeds with specific coat colours may have an increased prevalence of IBH. For example, black paint Shetland Pony mares were found to have an increased risk of IBH when compared with bay mares [16]. However, both Steinman et al. (2003) [17] and Grevenhof et al. (2007) [18] had previously found that coat colour had no significant effect on IBH incidence.

Nevertheless, it remains to be determined whether these apparent breed differences are due to genetic factors, environmental factors or a combination of the two, and whether there are specific genes that contribute to disease susceptibility. In a Dutch study, Grevenhof et al. (2007) [18] found that specific habitats in combination with warm, dry weather increased the prevalence of IBH. Similarly, Vychodilova et al. (2013) [19] found that the presence and expression of IBH can be influenced by many non-genetic factors, including the degree of exposure to insect bites, seasonal variations and climatic variations between years. Concomitant health conditions may also have an effect, and Kehrli et al. (2015) [20] demonstrated that occurrences of IBH were increased in horses that suffered from recurrent airway obstruction (RAO) compared to healthy individuals.

Few studies have been published on how to reduce the risk of IBH through selective breeding. At present there is no evidence of a simple dominant or recessive mode of inheritance, which makes selective breeding difficult to achieve, as there is uncertainty as to what genes to selectively breed for. Furthermore, IBH could be difficult to eradicate in breeds where there is a high prevalence of IBH but only a small subsection of the population with a high degree of inbreeding. Schurink et al. (2012) [14] has suggested that increased knowledge of the genes associated with IBH will contribute to our understanding of its biology, enabling more efficient therapy, prevention and selection to decrease IBH prevalence.

### 1.2. Management Strategies

IBH has the ability to cause a horse a significant level of pain and discomfort, and is therefore a welfare concern. Control of IBH is difficult due to the complex interplay of both hereditary and environmental factors in its pathogenesis [21] and its tendency to get progressively worse in succeeding years [22]. Schaffartzik et al. (2012) [1] therefore emphasize the importance of prevention of IBH rather than cure, as at present there is no definitive or simple treatment available. Schurink et al. (2013) [16] and Rendle (2014) [4] agree that a focus should be given on reducing the exposure to, and the biting of the horse by, *Culicoides* in order to alleviate any potential suffering and optimize the animal’s health, welfare and wellbeing.

Marsella (2013) [23] suggests three key points for the management of IBH: control of the itch to prevent self-trauma; the resolution of secondary infections; and the prevention of additional midge bites. Baker et al. (2015) [24] advocates a combination of physical barriers and chemical repellents as the most effective way to control IBH. This combination strategy, however, requires high compliance from the owner to maintain these labour-intensive measures over prolonged periods [4]. Schurink et al. (2013) [16] suggests that sensitive horses should be moved to low-risk habitats, or the current habitat adapted to reduce exposure to the *Culicoides*. Rendle (2014) [4] advocates keeping affected horses in open, windy locations, away from wooded areas and standing water where *Culicoides* spp. are likely to live and breed. Moreover, affected horses should be stabled at dawn and dusk especially in hot and humid conditions, as this is when *Culicoides* are most active [4,21].

Once stabled, measures can be taken to minimize the entry of the biting midge. Mosquito netting can be used to seal stable doors and windows to minimize *Culicoides* entry into the stable environment, thus preventing bites [4]. Rijt et al. (2008) [5] investigated the use of mosquito netting and found most midges were trapped at sunset when they were most active. A study by Baker et al. (2015) [24] further looked into the use of stable screening, and suggested that this management strategy should be implemented as part of an integrated control programme. Moreover, Braverman (1989) [25] suggested the use of fans in animal housing as a management strategy for discouraging adult *Culicoides* movements. However, Meiswinkel et al. (2000) [26] found that ceiling fans did not seem to suppress to any great extent the number of *Culicoides* captured indoors, suggesting that fans may be of limited effectiveness for control within the stable environment.

Chemical repellents and insecticides are a possible route for *Culicoides* control by either reducing the biting rate or reducing the *Culicoides* population size [27]. Many products are available on the market for the management of IBH in the horse; however, the effectiveness of each has been questioned. Baker et al. (2015) [24] found significant variation in the effectiveness of different commercially available insecticide-based treatments. Pyrethroid-based insecticides currently licensed and commercially available in the UK market have been found to cause 100% mortality in exposed *Culicoides* up to two weeks post-treatment in World Health Organization (WHO) cone bioassays [24]. From this finding it has been proposed, for example, that horse rugs should be treated with pyrethroid fly sprays as a management strategy for bite prevention [21]. Marsella (2013) [23] suggested that insect repellents could be applied, which contain at least 2% of permethrin. A study by Raat et al. (2008) [28] supports this, and found that application of an insecticide pour-on containing 3.6% permethrin reduced the number of *Culicoides* midges, however, the difference was not statistically significant. Alternative spot-on formulations or sprays can be applied all in a bid to prevent additional bites. Many of the available products require regular reapplication to be effective [4,23], which is labour-intensive for the owner.

Marsella (2013) [23] recommends immunotherapy as a treatment option over the longer term, however Wilson et al. (2001) [29] found that for IBH the reactivity to such a wide range of proteins complicates potential treatment of this condition by immunotherapy. Additionally, Hellberg et al. (2006) [30] found that each horse has a unique pattern of antibody binding, so specific immunotherapy with recombinant salivary gland allergens will vary from horse to horse. This requires immunotherapy to be tailored to each individual case.

Fatty acid supplementation has been proposed as a strategy for treating IBH with varying levels of success. A study by O’Neill et al. (2002) [31] suggested some benefit in using flaxseed to mitigate the skin test response to *Culicoides* extract from hypersensitive horses, whereas Friberg and Logas (1999) [32] found that linseed oil supplementation had no effect on the level of pruritus observed between the group supplemented and the controls. Topical therapy, systemic glucocorticoid and antihistamines are all available for IBH prevention, but only work as a preventative before the beginning of the allergy risk season, and with limited therapeutic success [23]. Peterson (2009) [21] did however suggest that although antihistamines are generally ineffective as a sole treatment, they are worth using for milder cases. Secondary infections resulting from the self-trauma associated with IBH can be resolved via a combination of topical antimicrobial therapy and systemic antibiotics for more severe cases [23].

Potential new therapeutic approaches to manage IBH have also been explored. Anti-IgE therapy has been shown to have some success in the treatment of human atopic eczema [33], and this may be beneficial for IBH. Furthermore, Jonsdottir et al. (2016) [34] found that intralymphatic administration of small amounts of pure *Culicoides* allergens can induce a high immune response that could be a positive immunotherapy approach against IBH, however, trials to investigate this further are required to confirm this as an appropriate and effective management strategy.

From this short overview of the literature, it is evident that management and treatment of IBH is challenging. All available approaches to IBH control have limitations, emphasizing that control plans must be tailored to the individual situation, and may involve some adaptation. This qualitative research project is an exploratory pilot study, set up with the aim of producing indicative findings about the impact of IBH in horses from the perspective of different equine stakeholders. The objectives were to gain different stakeholders’ knowledge and experiences with IBH, and to assess the current management strategies used to control midges and to treat the condition when it occurs.

## 2. Materials and Methods

### 2.1. Qualitative Methods

Using semi-structured interviews as a qualitative approach to research is beneficial as it enables an in-depth understanding to be gained on a topic through the opinions of different participants [35]. Each response is unique to the individual through open questions that require a detailed response to be given, but when multiple interview responses are analyzed, patterns emerge. Having face-to-face interviews has the added benefit of providing social cues such as body language that provide extra information to the verbal response [36]. Furthermore, synchronous communication is achieved with the researcher and the participant by reacting off one another, and can result in more spontaneous responses being achieved [36].

An integrated approach to collecting and using qualitative data to investigate health problems in veterinary medicine is increasingly being recognized [37,38,39]. Even with smaller sample sizes compared to quantitative research, the findings of qualitative research are often indicative of the beliefs and experiences of the wider population from which the sample is drawn [40]. Christley and Perkins (2010) [41] suggest the benefits of qualitative research for veterinary research, especially on topics that are poorly understood, and to find out what is important to veterinary practice clients. Upjohn et al. (2013) [42] believe that ‘qualitative methods provide greater opportunity for the researcher to gain a detailed understanding of local issues, an appreciation of how owners defined and prioritized the problems affecting them and their animals, and their perception of the importance of issues being addressed by current intervention’.

Qualitative research methods are increasingly being used to investigate health issues in the farm animal sector [43,44,45,46,47], and have also been used in equine health and welfare research. Litva et al. (2010) [37] investigated perceptions of crib-biting behavior in horses, and Scantlebury et al. (2014) [48] used a mixed methods approach involving in-depth interviews and questionnaires to gauge the level of understanding, and approaches to the management, of equine colic by owners. Semi-structured interviews and focus groups were used to explore equine stakeholders’ views on horse welfare [49,50], and qualitative methods have also been used to investigate private veterinarians’ [51] and horse owners’ [52] attitudes to Hendra virus management in Australia.

The current published literature on IBH is based on epidemiological and laboratory-based scientific research, with an absence of qualitative investigation of IBH. Through this project, perceptions and experiences of IBH from different equine stakeholders were gained using interviews, gathering data that enables a richer understanding of the impact of the condition in the field.

### 2.2. Sampling Method

A combination of purposive, convenience and snowball sampling methods were employed. Purposive sampling is commonly used in qualitative research, with participants recruited based on their relevance to the subject area [53], while snowball sampling allowed the researcher to access participants through contact information provided by other participants [54]. The final sample of nine included participants who were involved in horse livery, riding schools or equine competition (5), hobbyists (3) and an equine veterinarian. The veterinarian was included to help triangulate the findings from the other stakeholders, and to offer an alternative perspective on IBH. Although nine is a relatively small sample size in qualitative research [55], the interviews allowed for the generation of enough data for thematic analysis to unearth trends and patterns [56], and this acts as a useful exploratory pilot investigation of the subject.

Initially, potential participants were identified from current contacts of the first author in the industry and through internet research of equine establishments in the West Midlands region of England. Once identified, a request form was sent to potential interviewees via email to ascertain whether they would be interested in participation in the study. Within this request form IBH was introduced along with the purpose of the study and the main aims and objectives that the research hoped to achieve. The individuals that responded to the initial request form were then invited to take part in a face-to-face interview lasting around 30 min at a time and place of their convenience.

### 2.3. Ethical Considerations

Prior ethical approval was granted by a research ethics review committee at Harper Adams University (approval No. 1116-201612-STAFF). Prior to the commencement of each interview, participants were provided with an information sheet that outlined the purpose of the study, and a consent form was read and signed by participants to confirm their willingness to participate and the interview audio-recorded. Participants were assured of anonymity in the study.

### 2.4. Interview Conduct and Analysis

A list of topic areas was devised prior to the interviews and agreed by both authors to ensure that sufficient information was gained to achieve the main aims and objectives of the research. The nine interviews were all conducted by the first author (university student studying an animals-related degree course), which provided a consistent interviewing approach. The semi-structured nature of the interviews was shaped by the flow of conversation and not completely by pre-determined questions, allowing for naturalistic enquiry. However, the use of a list of topic areas ensured that the researcher covered all relevant areas of enquiry in around 30 min. Each interview began with some initial questions about the participant themselves and their involvement with horses, followed on by more specific questions regarding their opinions, knowledge and experiences of IBH. Initially, two pilot interviews were conducted to ensure that the interviews ran smoothly, and to allow for appropriate amendments to be made based on the feedback received.

The interviews were transcribed using f4 transcription software (www.audiotranskription.de) and analyzed using NVivo qualitative data analysis software (Version 11, QSR International Pty Ltd., Melbourne, Australia) for thematic analysis to identify trends within the data [56]. Thematic analysis is commonly used as an independent and reliable approach to qualitative analysis that aims to identify themes, patterns and organize ideas [57].

## 3. Results

### 3.1. Stakeholder Knowledge and Awareness of IBH

All participants were at least aware of IBH as a skin disorder of the horse. Of the nine, six had previously owned a horse with IBH, and one had no previous personal experience with the condition. The most significant response when asked about the signs associated with IBH was the fact that the horse would be pruritic. Although initial awareness and knowledge of IBH was generally high, when discussing the aetiology of IBH and *Culicoides* midges in more detail, knowledge varied. Although the majority of participants were aware that IBH is caused by an insect, some were unaware of more specific details in relation to the fact that it is mainly caused by *Culicoides* midges:

‘I wouldn’t have known necessarily that it was a midge before now, and I think that that is probably quite similar to a lot of people.’

(Int 1, riding school employee/hobbyist)

‘The cause—I’m not 100% sure. I assume it’s some sort of autoimmune response to horse fly bites.’

(Int 8, hobbyist owner)

‘Is it just like a midge? I don’t know how it causes sweet itch - just that it’s kind of an irritant, that they want to itch and rub their skin.’

(Int 2, racing yard employee)

When asked about the conditions that the *Culicoides* midge favors, there appeared to be uncertainty amongst some participants:

‘I don’t really know, is it like damp trees and things like that?’

(Int 2, racing yard employee)

‘I’m not sure, but I would say in warm and wet areas they tend to breed and they tend to swarm.’

(Int 7, hobbyist owner)

Others had a deeper level of knowledge on the environmental conditions that favour midges and their habitat, as demonstrated by these quotes:

‘Midges usually prefer the warmer weathers during the summer and places where it is wet like ponds or standing water in the field. Usually once we get a good frost the midges die off.’

(Int 6, livery yard employee)

‘It favours being anywhere near stagnant water, it’s even worse if you turn them out and you have hedges, as they live in hedges. If you have a very wet summer where you have water sat on hard ground they are then attracted to the damp conditions. They don’t survive in the winter—in summer they arrive.’

(Int 3, competition owner)

### 3.2. Impact of IBH on Horse Owners

When looking at the effect of IBH on daily routine there was a clear difference between equine stakeholders who had experienced IBH through horse ownership and working with horses, compared with an equine stakeholder with no prior experience of the condition. When participants were asked on whether having a horse with IBH would affect their daily routine, a participant who had no prior experience with IBH stated that:

‘I don’t think it would massively affect your daily routine.’

(Int 5, hobbyist owner)

However, the other eight participants said that IBH would affect their daily routine, although the impact varied between participants and the severity of the IBH they had encountered:

‘It’s just an extra step, like any rugs or sprays or anything like that—it doesn’t take too much of your time to add on.’

(Int 8, hobbyist)

‘It should have at least two visits a day which will take up a lot of time and then there is the time you will spend having to apply various lotions. If you stable the horse then you will need to spend time mucking out. Sweet itch happens in the summer when most horse owners would be enjoying having horses turned out and possibly not needing to visit so often.’

(Int 6, livery yard/riding school employee)

‘You’ve got to work your whole routine around that particular pony as its best to have the pony out in the middle of the night and back in before the midges come back out or only have them outside in the middle of the day so this really does impact when you’ve got to see to them. Also it does increase your costs because the best thing to do is before the midges start in early spring is to cover them up with fly rugs, sweet itch rugs etc. which is obviously extra costs.’

(Int 4, competition owner)

All participants were in agreement that having a horse that has IBH can have limitations on what can be done with them. Participants made particular reference to showing being difficult to do with a horse that suffers with IBH:

‘From a showing point of view it would definitely affect having the animal as you couldn’t actually use it for the purpose that you have got it for. Other disciplines it probably wouldn’t affect as much.’

(Int 4, competition owner)

‘I think showing is definitely off the cards because like the slightest bit of anything, even if it is a scar in the showing world, that is unacceptable.’

(Int 1, riding school employee/hobbyist)

‘Yes I would say so, especially in showing … If their mane and tail is scabby and sore, I think you would lose marks for the condition of the horse, even though sometimes you can’t help it.’

(Int 2, racing yard employee)

There was a mixed consensus when discussing the consideration of future purchase of a horse with a history of IBH. The participant with no prior experience of the condition said that they would not be deterred from purchasing a horse with a history of IBH, in contrast to others who had experienced it:

‘Yes definitely, because it stops you doing so many things. They constantly look scruffy, and also the management time that is involved.’

(Int 1, riding school employee/hobbyist)

‘Yes, because of the time and money involved in a horse with sweet itch.’

(Int 6, livery yard employee)

Participants said that such a purchase decision was also dependent on other individual circumstances:

‘It depends, because the ground I have my horse on is quite wet, so you know you’re going to exacerbate the problem if they have it quite severe.’

(Int 7, hobbyist owner)

‘It’s tricky because I tend to buy them at a young age and as I have said earlier it doesn’t come through until they are older so it would put me off, but at the same time if the animal was good enough I know I could manage it.’

(Int 3, competition owner)

All participants with experience of IBH were in agreement that management incurred additional financial costs in addition to the time costs:

‘It does increase your costs because the best thing to do is before the midges start in early spring is to cover them up with fly rugs or sweet itch rugs which is obviously extra costs.’

(Int 4, competition owner)

‘Obviously it takes more time if you are trying to wash it or you’ve got other things to do with it and going to the vet—that’s the big cost.’

(Int 2, racing yard employee)

‘There is the cost of extra rugs which are very likely to need replacing frequently due to rips from the horse itching. If stabling the horse there is the cost of hay and bedding. And then there is the cost of the vets for the cost of steroids, antihistamines and benzyl, which will possibly be needed on a long-term basis.’

(Int 6, livery yard/riding school employee)

### 3.3. Management Strategies for IBH

As there is no cure for IBH, management of the condition is essential. The veterinarian supported this concept:

‘A lot of it is management really, that is what I recommend to people as it is the best way to deal with it.’

(Int 9, veterinarian)

The management strategies that participants had implemented were found to often involve an integrated approach of both physical barriers and chemical repellents:

‘Fly rug on at all times when he didn’t have his winter rugs on. I had some creams that I would put on his skin as well and I would use fly sprays.’

(Int 1, riding school employee/hobbyist)

‘Covering up and rugs, there are also creams and products available that can be applied to help to alleviate it, also fly repellents.’

(Int 4, competition owner)

‘Have used the methods of fly rugs, stabling and applying lotions daily. If there was ever a sweet itch vaccination then I would definitely give it a try.’

(Int 6, livery yard/riding school employee)

Six of the participants took midge avoidance into account as a preventive management strategy to control IBH. This midge avoidance was by either stabling at time of the day when midges were most active, or moving horses into an area where midges were less likely to breed and inhabit:

‘Keeping them in an area where you are less likely to encounter midges or possibly stabling the horse in the early hours and in the evening.’

(Int 7, hobbyist owner)

‘Find some stables that are away from any sort of standing water or even rivers it would be best if you could stay away from those.’

(Int 9, veterinarian)

All nine equine stakeholders were in agreement that the use of an insect rug was an effective management strategy to act as a physical barrier to the midge so they could not bite the skin of hypersensitive horses:

‘Fly rugs obviously help because they stop them getting to the skin in the first place.’

(Int 1, riding school employee/hobbyist)

‘During the summer months he has to be in a fly rug.’

(Int 6, livery yard employee)

The veterinarian supported the use of rugs as the most effective management strategy, but rugs brought their own challenges in terms of cost and practicality:

‘I always tell people that the best way is a lot of the time to buy a cover-all sweet itch rug that acts as a physical barrier … The lady I saw recently has actually done this and uses that, and she has had great success with it … A full-on specific midge sheet you are probably looking at £100–£200, which is quite a lot of money, particularly if your horse is prone to tearing them up.’

(Int 9, veterinarian)

‘Routine-wise, it’s quite hard when it’s red hot to ask a horse to wear a fly rug. It’s probably actually easier to have them out during the night when it’s cooler, and have them in during the day.’

(Int 3, competition owner)

Three participants also reported the use of dietary supplementation in a bid to manage IBH:

‘I fed him garlic as I [was] told that that helps to deter flies and midges from biting, so that’s for him.’

(Int 1, riding school employee/hobbyist)

‘Supplement with vitamin B12 and Marmite is also supposed to be a good one as well.’

(Int 7, hobbyist owner)

‘During the summer he is fed a global herbs supplement to try and help him.’

(Int 6, livery yard employee)

To relieve the symptoms of pruritus, some of the owners administered antihistamines, with some reported success, and this approach was also supported by the veterinarian interviewee:

‘I give them antihistamines—personally I have a friend who understands drugs and they are perfectly healthy to give to horses. I give a ratio of 450–500 kg of horse about five to six tablets a day—this I think relieves them of the need to itch. I know a friend who had a filly that suffered very badly with it. She sought help from her vet—her vet prescribed [antihistamine] that helped but didn’t cure it.’

(Int 3, competition owner)

‘In both severe cases the ponies were stabled overnight and only out for a few hours in an electric fenced area where we removed anything that might enable them to itch. They were also hogged and tail pulled and tuned out in fly rugs. Antihistamines were given, and benzyl benzoate, [and] fly sprays applied daily.’

(Int 6, livery yard/riding school employee)

‘Yes I would go with the use of antihistamines, maybe for the mild cases.’

(Int 9, veterinarian)

Corticosteroid injections or creams were also sometimes used as a management strategy for IBH:

‘I started a course of injections that did help short-term, but financially it was crippling.’

(Int 3, competition owner)

‘If the case is severe then, yes, the vet has a role, such as coming out to give steroid injections.’

(Int 6, livery yard/riding school employee)

‘If they are really bad and they have got to the stage where they are rubbing like crazy, then we would look into the use of steroid cream to try to calm the skin down.’

(Int 9, veterinarian)

Overall, there was an evident sense of frustration amongst some stakeholders about how successful management of IBH could actually be in the field:

‘I don’t think that they are actually that effective. I don’t think people know, I’m not saying they don’t know what causes it, just that I don’t think they know how to stop it. From what I know, the [cream] I had for Dodge was supposed to be (according to every review) supposed to be the one that worked the best, and it still didn’t stop him ripping his mane out.’

(Int 1, riding school employee/hobbyist)

‘I think once they are prone like it will come back there is no way really of stopping it.’

(Int 2, racing yard employee)

### 3.4. Impact of IBH on Equine Welfare

From a welfare perspective, all interviewees were in agreement that an equine with IBH had compromized welfare and wellbeing leading to irritation and suffering, at least to some degree. These quotes are examples of their responses when asked about this welfare impact on the animal, and whether suffering was involved:

‘I think definitely it impacts their welfare—it’s very pruritic, or seemed to be anyway when she did get it. When the flies were around she seemed to be very uncomfortable. So I think from their point of view it is a much bigger issue than it is from our point of view.’

(Int 8, hobbyist owner)

‘Yes, I would say definitely it compromises the horse’s welfare if it is just left.’

(Int 9, veterinarian)

‘I’ve known ponies that will go over electric fences or through it to rub.’

(Int 3, competition owner)

‘Yeah if it’s unmanaged then yes, like I know I think that the worst one out of all of ours was the Shetland and the fact that he had to live out because he wouldn’t stay in—I think he suffered quite a lot, I would say that. His skin was so bad, scabs were everywhere and he was so uncomfortable that he was actually suffering even though we tried to do everything we could for him—like I would say he was still suffering. I think the milder ones as long as you are managing it, it’s okay, but if you don’t manage it they do definitely suffer from it it’s not nice for them.’

(Int 1, riding school employee/hobbyist)

‘If not kept on top of, or not treated at all, then sores can become infected and it’s likely horses can become very depressed.’

(Int 6, livery yard/riding school employee)

From a behavioural perspective, two of the interviewees believed the itching of the horse became a learned behaviour that they would perform initially in response to the hypersensitivity, but could continue even after the condition was sufficiently managed:

‘They’re covered up 24/7, and never given a chance to actually start to rub, because I do think it’s learnt behaviour.’

(Int 3, competition owner)

‘Some of it with the horse they get so itchy and so used to the fact that even if you are soothing it (the itch) they carry on itching because it’s sort of habitual.’

(Int 1, riding school employee/hobbyist)

### 3.5. Veterinary Involvement in Management of IBH

Despite the obvious welfare impact on horses, and the frustrations for owners in managing IBH, there appeared to be a general reluctance to involve veterinarians in advice and treatment concerning IBH. One owner thought this was because the condition was perceived as a common condition which owners felt they could manage by themselves:

‘I think because it is a well-known issue, I don’t think people do contact the vet. I’ve never contacted a vet over it because you sort of google it, you research it and it’s something that comes up with loads of different, people have loads of different ideas on how to manage it. The horses aren’t ill, they’re not lame or anything like that, so you don’t feel like you need a vet but you can sort of manage it yourself. It’s not a life-threatening problem—they are basically just itching, there’s so many, so many treatments and remedies out there to try and help it that you go through all of them first and then by the time you have done all them you sort of just accept it as something that is rather than taking it to the vet and I think yeah, the vet isn’t something you should go to for something like sweet itch.’

(Int 1, riding school employee/hobbyist)

This stakeholder perception was supported by the veterinarian, who suggested that because of the multiplicity of possible management and treatment strategies, owners tended to use a trial and error approach to finding a successful combination that worked for them:

‘Yes definitely, I think there is always the temptation to try and manage it yourself, especially if you don’t know any better, and just try loads of different fly repellents and not really know the management changes like not turning them out in the morning and evening when they (the midges) are most active.’

(Int 9, veterinarian)

Veterinary involvement, as far as several of the owners was concerned, was likely only in severe cases of IBH, and when self-management had failed:

‘Yes, we contacted the vet for the pony because it was that severe.’

(Int 2, racing yard employee)

‘I would only approach a vet if it got really bad—there are a lot of creams and that available that you can use to manage small rashes, and if it got really nasty I would probably use a vet if they needed antibiotic ointment or something.’

(Int 5, hobbyist owner)

‘If the case is severe then yes the vet has a role such as coming out to give steroid injections and prescribing medication, or if it’s the first time owning a horse with sweet itch, then a visit from the vet would be useful for the owner.’

(Int 6, livery yard/riding school employee)

‘I think it would depend on the severity really. If it was mild then I would probably just manage it on my own like I do, but if it was more severe and the horse really was showing signs of distress and discomfort, then I would definitely go to a vet for advice.’

(Int 7, hobbyist owner)

Veterinary treatment was viewed negatively as an additional cost, and owners seemed to prefer attempting to manage the condition on their own, despite the desire of the veterinarian to be involved at an earlier stage:

‘I think people always worry with vets that we will charge them loads of money … I think a lot of it is just pushing people to come and see us … If they are having problems they can come and talk to us, because a lot of people tend to just ask around the yard and come to the vet later, and we say we could have caught this slightly earlier if you had just come to us and had a chat. People just don’t always want to go to the vet in case we charge them shedloads of money.’

(Int 9, veterinarian)

### 3.6. Further Research and Stakeholder Information on IBH

Some of the participants thought that IBH was kept under the surface and seen as a ‘taboo’ subject within the equine industry:

‘When you hear someone say a horse has got sweet itch, people automatically back off, and if people understood it a bit better they might not be so deterred.’

(Int 8, hobbyist)

‘I also think that it’s a bit of an ‘elephant in the room’. I think that if you’re a horse owner and you’ve owned ten horses, three or four have probably had it, and you wouldn’t admit it.’

(Int 3, competition owner)

Despite this apparent reluctance to admit IBH, all participants thought that more information being available would be helpful to improve stakeholder awareness of the condition:

‘Yes, more information would be useful to understand the disease more.’

(Int 5, hobbyist owner)

‘I think leaflets being more available would be good, and also online information, as everyone uses online for pretty much everything if they are not sure on it. I would probably look online as there [are] always forums on what other people have done.’

(Int 2, racing yard employee)

A competition owner agreed that more support from veterinarians would be useful, but had a caveat, suggesting that veterinarians could offer treatment that was not justified:

‘Equine vets should offer annual or six monthly talks to help those owners who are a little bit lacking in knowledge because they have never had to deal with it before, so yes, they do have a role to play. But they are, well, they almost jump in feet first and try and offer you care that is not actually needed for a sensitivity rather than for pure sweet itch.’

(Int 3, competition owner)

The veterinarian acknowledged the need for more information to increase knowledge on IBH, and identified a possible gap in his own practice policy on the condition:

‘[Information leaflets]—it’s not something we currently offer, but I mean if I thought somebody needed more information on sweet itch I could direct them on this. It would be a good thing to have this information available, as it is a relatively common problem. The more that people know about it, the more that it can be spotted earlier on.’

(Int 9, veterinarian)

From these findings it would appear that participants were in agreement that more needs to be done to raise awareness and knowledge of IBH within the equine industry. Ongoing research was also viewed as desirable to provide future developments in the control of IBH:

‘I think it would be good if vets provided more information and if more research is done so that they know what could alleviate it and prevention is the best way to deal with it. Also they’ve never really found a cure but obviously if there was a lot more research this could be achieved … A cure would be very helpful to the horses that are suffering with it, so if they can find a cure that would be absolutely marvellous.’

(Int 4, competition owner)

‘Yes, I think that probably the more research that is done, and the more support that is available by vets, would be better, as obviously it is something that can be really detrimental to the horse.’

(Int 1, riding school employee/hobbyist)

## 4. Discussion

This qualitative approach provides an understanding of the key issues equine stakeholders felt were associated with IBH, and gives an appreciation of the relative importance of these issues. As such, it begins to address a gap in the literature for this condition. The study is limited by the relatively small number of participants, meaning that the results should be viewed with some caution, but the authors believe them to be valuable nonetheless. Future research could use an increased number of interview participants or a questionnaire of a much larger sample to gain a wider appreciation of opinions and experiences with IBH in horses from different stakeholders across the equine industry. The interviews were all conducted in the West Midlands region of England for logistical and financial reasons, and it would therefore be useful to conduct research in other areas of the United Kingdom to investigate whether these indicative findings are generalizable at a national level.

All equine stakeholders had some knowledge of IBH, but only those with personal experience of the condition had a more advanced understanding of it. All nine respondents reported at least an awareness of sweet itch, and the majority had personal experience in their horses in the present or past. All referred to the pruritic nature of the disorder, and this theme recurred throughout the interviews, suggesting that participants placed a high degree of importance on the presence of pruritus in identifying IBH. This finding is in line with Rijt et al. (2008) [5] and Hellberg et al. (2009) [3] who also referred to the pruritic nature of the condition as the most prominent clinical sign, and Peterson (2009) [21], who suggested the pruritic nature of the condition as a means of diagnosing IBH.

In general there appeared to be a lack of specific knowledge across the majority of participants that IBH is caused by a hypersensitivity reaction to the saliva of the *Culicoides* biting midge. Some participants did recognize that the *Culicoides* midge is likely to be more present in areas with water present, and that they are more active during dawn and dusk [4]. *Culicoides* midges are also recognized as poor fliers, making horses less exposed to midge bites in windy, open areas, however this was only mentioned once across the interviews, suggesting that it is not commonly known amongst equine stakeholders. These findings suggest that although the scientific research is available on the *Culicoides* midge, it is for the most part not being accessed or utilized by horse owners to guide their management strategies for IBH. This is perhaps surprising considering that the majority of participants had personal experience of IBH in horses they had owned or worked with. This suggests a gap in specific knowledge in relation to the *Culicoides* midge that needs to be addressed to ensure that equine stakeholder knowledge of the aetiology of IBH is better understood to allow for more effective prevention and management of the condition.

The difference in the extent to which the participants felt that daily routine would be affected could be due to their level of previous experience with the condition, and also suggests that it is based on individual variation of IBH manifestation between horses. Hellberg et al. (2006) [30] supports this possibility, finding that each horse has a unique pattern of antibody binding, which could affect case severity. Overall, in terms of the management of IBH it was obvious that there were many different strategies available, and no consensus on what was best for a horse suffering from the condition. A lot of what seems to be done is based on what the individual had found to be effective in previous cases, or what other horse owners had recommended to them. The effectiveness of each strategy seemed to be dependent on individual circumstances and the severity of the case. The financial and time cost implications of managing IBH-affected animals may be one of the reasons associated with why some participants with previous experience with IBH would be wary of purchasing a horse in the future with a previous history of IBH.

Furthermore, this research highlights the need to optimize horse welfare through education and knowledge exchange between stakeholders, veterinarians and professional representative bodies. What is significant is the general lack of veterinary involvement reported by these stakeholders, despite their frustrations about the condition and its impact on their horses. Instead, there was almost a resigned acceptance of the condition as a daily part of horse ownership in the spring and summer seasons, and a sense of managing as best they could, only involving veterinarians if the condition was deemed severe enough to merit the additional cost. It was clear that IBH can be a significant recurrent cause of suffering for susceptible horses.

All equine stakeholders felt that the provision of more information on IBH would be useful. A single set of recommended guidelines on IBH that provided scientific information could be beneficial. This would not only increase knowledge of the condition across the industry, but could also make individuals better prepared when it arose in the future. These guidelines could be supported by veterinarians and professional representative bodies to reinforce that the information is from a trusted source and based on published scientific findings. Moreover, annual talks and discussion groups provided by practice veterinarians and research scientists would be useful to keep stakeholders informed and up-to-date with developments in IBH research. A focus therefore needs to be on collaboration between research professionals and stakeholders in the industry to manage IBH effectively, optimize equine welfare, and improve the compliance of horse owners in the often costly and labour-intensive management that the condition requires, with more emphasis on prevention rather than treatment.

## 5. Conclusions

The findings from this study provide a useful starting point for the qualitative investigation of the impact of IBH in the field, but any conclusions drawn should be made with caution due to the small sample size and limited geographical area of research. Further research is required on equine stakeholders’ perceptions and opinions on IBH so that more generalizable conclusions can be drawn. The participants’ clear awareness of the often-serious welfare implications that can result from the condition reinforces the importance of effective management of IBH from an equine welfare perspective.
